# Magnetic perovskite nanohybrid based on g-C_3_N_4_ nanosheets for photodegradation of toxic environmental pollutants under short-time visible irradiation

**DOI:** 10.1038/s41598-023-48725-x

**Published:** 2023-12-03

**Authors:** Soheila Sharafinia, Abdolhadi Farrokhnia, Ensieh Ghasemian Lemraski, Alimorad Rashidi

**Affiliations:** 1 Department of Chemistry, Faculty of Science, Shahid Chamran University of Ahvaz, Ahvaz, Iran; 2 Department of Chemistry, Faculty of Science, Ilam University, Ilam, Iran; 3 Nanotechnology Research Center, Research Institute of Petroleum Industry (RIPI), Tehran, Iran

**Keywords:** Environmental sciences, Nanoscience and technology

## Abstract

In this study, a magnetic perovskite nanohybrid based on g-C_3_N_4_ (gCN) nanosheets was synthesized and developed for the efficient photodegradation of toxic environmental pollutants under short-time visible irradiation. The synthesis of this nanohybrid involved the incorporation of SrTiO_3_:N (STO:N) and ZnFe_2_O_4_ (ZnF) onto the g-C_3_N_4_ nanosheets through a simple reflux method. Our investigation encompassed a comprehensive suite of analytical techniques, including BET, TGA, TEM, SEM, EDX, DRS, VSM, XRD, photocurrent, and FT-IR, to elucidate the physicochemical characteristics of this nanocomposite in the context of its application in photodegradation processes. The nanohybrid displayed significantly enhanced photocatalytic activity compared to its individual components, achieving a degradation efficiency of over 90% for various pollutants, including organic dyes like Rhodamine B (Rh-B), within a short irradiation time. This enhanced activity can be attributed to the synergistic effect between gCN, STO:N, and ZnF, which promotes the generation of reactive oxygen species and facilitates the degradation process. Notably, the nanocomposite containing 20 wt% STO:N perovskite and 20 wt% ZnF demonstrated the highest Rh-B degradation rate under visible light irradiation within just 30 min. Furthermore, the nanohybrid displayed excellent stability and reusability over seven consecutive runs, retaining its high photocatalytic activity even after multiple cycles of degradation. This remarkable performance can be attributed to the strong interaction between the gCN nanosheets and the magnetic perovskite components, which prevents their aggregation and ensures their efficient utilization. Additionally, the nanohybrid exhibited excellent visible light absorption, enabling the utilization of a wider range of light for degradation. This feature is particularly advantageous, as visible light is more abundant in sunlight compared to UV light, rendering the nanohybrid suitable for practical applications under natural sunlight. In conclusion, the ternary gCN-STO:N@ZnF nanocomposite represents a promising candidate for the treatment of organic pollutants in aqueous environments, offering a versatile and efficient solution.

## Introduction

In recent years, we have witnessed the emergence of problems such as global warming, decreasing the amount of energy required, and increasing environmental pollution, which is due to the ever-increasing growth of the human population and the development of the industry^1^. Therefore, the demand for use of clean energy and the removal of environmental pollutants has become the focus of many research communities^2^. Various strategies, such as chemical^3^, physical^4^, and biological^5^ methods, are used to remove pollutants. Among these methods, the major emphasis is on photocatalytic processes, especially photocatalysts based on semiconductors. In recent years, photocatalytic technology has become a suitable method for degrading dye pollutants (for example, azo dyes)^6,7^. Semiconductor solid oxides that are activated under light are called photocatalysts. Photocatalysts, after absorption of light and activation, their electrons are excited to the conduction band, and holes remain in the valence band. The resulting holes and electrons are strongly oxidizing and reducing, respectively^8,9^. The electron/hole (e^−^/h^+^) pairs produced react with the H_2_ and O_2_ molecules on the surface of the particles, caused to the removal of contaminants via the production of radicals. Graphite nitride carbon (gCN) is a polymeric semiconductor with an energy gap of 2.7 eV and responds well to visible light (up to 460 nm). Medium energy gap, low cost, simple preparation method, high chemical stability, and non-toxicity make gCN suitable for photocatalytic applications, such as degradation of organic pollutants such as azo dyes under visible light^10,11^. Despite the remarkable electronic and optical properties of gCN, its faces limitations such as high rechargeability of cargo carriers, small specific surface area, low conductivity, and low capacity (VB) potential. One of the methods used to increase the photocatalytic activity of gCN is metallic and non-metallic doping^12,13^. In recent years, gCN has been widely used to form heterogeneous connections with perovskites to improve their photocatalytic performance. Heterogeneous bonds are mainly formed by gCN and^14^ LaTiO_3_/N-LaTiO_3_, LaFeO_3_^15,16^, CaTiO_3_ and SrTiO_3_ doped with N_2_^17^. Strontium titanate (SrTiO_3_) is a perovskite with a cubic structure. The crystal structure of SrTiO_3_ consists of an octagon TiO_6_ in which the cations Sr^2+^ are in the octagon, Ti^4+^ is located between the oxygen atoms in the octagon system^18^. High photocatalytic activity and optical and chemical stability compatibility are the prominent properties of these titans broadly applied in different applications such as a bioelectronic, actuator, photonics^19,20^ multilayer capacitors, and others. The SrTiO_3_ photocatalyst is a highly efficient perovskite for inactivating bacteria, producing H_2_ fuel from water and pollutants degradation^21,22^. The SrTiO_3_ perovskite is activated only under irradiation of UV light. Due to the dangers of UV light to living, it is not appropriate to use this light. Also, because less than 4% of sunlight is UV, it is economically viable to use it as a light source. Although SrTiO_3_ has a favorable absorption edge potential for a free radical generation; but, pure SrTiO_3_ has a bandgap of 3.25 eV and is activated through UV irradiation^23^. Therefore, by reducing the SrTiO_3_ bandgap, it can be used in the visible area. However, it is necessary to reduce the energy gap of SrTiO_3_ to the visible region^24^. Adding metallic and non-metallic impurities to these perovskites is a good way to reduce their energy gap^25^. Since non-metallic doping does not produce phase impurities, doping SrTiO_3_ with N is a suitable method to reduce its bandgap to the Vis area^25^. A convenient and efficient method for synthesizing this type of photocatalyst is sol–gel. The sol–gel method can achieve various advantages such as easy doping ability, industrial scale production, synthesis at low temperatures, and production of a highly homogeneous material. Moreover, adding magnetic materials to the composites can solve problems for instance separation and regeneration ability in the photocatalytic procedure. The zinc ferrite semiconductor (ZnFe_2_O_4_) photocatalysts have received much attention due to their advantages such as easy fabrication, low cost, and suitable energy gap in the visible light region (1.9 eV) as high photo-chemical stability. The gCN-STO:N@ZnF nanocomposite boasts a range of merits for degradation processes:

i. *Enhanced photocatalytic activity* This nanocomposite, comprising g-C_3_N_4_, SrTiO_3_:N, and ZnFe_2_O_4_, exhibits significantly improved photocatalytic activity when compared to its individual components. This heightened activity results in more efficient degradation of pollutants and organic compounds.
ii. *Visible light absorption* The nanocomposite's capacity to absorb visible light, owing to the presence of g-C_3_N_4_ and ZnFe_2_O_4_, broadens the spectrum of light available for degradation. This advantage is especially noteworthy since visible light is more abundant in sunlight, as opposed to UV light.
iii. *Stability and reusability* This nanocomposite demonstrates remarkable stability and can be easily separated from the reaction mixture post-degradation. This characteristic permits its reuse in subsequent degradation processes, thereby reducing the need for additional catalysts and minimizing waste production.
iv. *Wide range of applications* The gCN-STO:N@ZnF nanocomposite can be effectively utilized in diverse degradation processes such as wastewater treatment, air purification, and soil remediation. Its versatility renders it suitable for a variety of environmental applications. In summary, the gCN-STO:N@ZnF nanocomposite offers distinct advantages, including enhanced photocatalytic activity, visible light absorption, synergistic effects, stability, reusability, and wide applicability. These qualities make it a promising catalyst for a wide range of degradation processes.

Physico-chemical specification of the synthesized photocatalysts were investigated by some methods for example Fourier transform infrared (FT-IR), X-ray diffraction (XRD), UV–Vis emission reflection spectroscopy (DRS), X-ray energy diffraction (EDX) analysis, transmission electron microscopy (TEM), scanning electron microscopy (SEM), and vibrational sample magnetometry (VSM).

## Experimental

### Materials

All the chemical materials used in this study, which are listed below, were purchased from Merck, Germany:

Hydrochloric acid (HCl99%), Rh-B, Dimethylformamide (DMF), Zirconium tetrachloride (ZrCl_4_), Benzene-1,4-dicarboxylic acid (Terephthalic acid (BDC), Melamine (C_3_H_6_N_6_, 99%), Zinc nitrate hexahydrate (Zn (NO_3_)_2_⋅6H_2_O), Iron nitrate nonahydrate (Fe (NO_3_)_3_⋅9H_2_O), Strontium nitrate (Sr(NO_3_)_2_), Sodium hydroxide (NaOH), Titanium tetra isopropoxide (TTIP-C_12_H_28_O_4_Ti), Ethylenediamine (C_2_H_8_N_2_).

### Instruments

Spectrophotometer (UV–VIS) Mini model (SHIMAZU), X-ray diffraction (EDX) field emission electron microscope (FESEM) model MIRA3 LMU MI2851376IR, Perkin Elmer Thermal Gravimetric (TGA), Surface Area Analyzer (BET) model PHS-1020 (PHSCHINA), Infrared spectrometer (FT-IR) model Perkin Elmer-spectrum 65, X-diffraction device (XRD) model. Broker AXS-D8 Advance, Vibrating sample magnetometer (VSM) model MDKF made by Kashan Daneshpajooh Magnet Company, Reflective scattering spectroscopy (DRS), Philips EM208S 100kV transmission electron microscope.

### Preparation of STO

For the preparation of 1, TTIP (0.27 g) was added to methanol (20 ml) as a solvent. The resulting solution was stirred for 10 min to form a homogeneous solution (solution (1)). In another beaker, strontium nitrate (0.2 g) was added to 1 ml of distilled water was stirred for 10 min (solution (2)). Solution (2) was then added to the solution (1) to obtain a white cell. The resulting cell was stirred with a magnetic stirrer for 10 min (to complete the hydrolysis reaction). Then, as a nitrogen source, ethylenediamine (0.08 g) was added to the solution and mixed for 5 min. Then, by adding sodium hydroxide (1 M), its pH was adjusted to 10 and stirred well. After 10 min, the temperature of the solution increased to 100 °C, and after 45 min, the white gel was obtained. The resulting gel was dried for 30 min to give a yellow powder. The powder is calcined for 2 h at a temperature of 500 °C^26^.

### Preparation of STO:N

For the synthesis of STO:N nanoparticles, all the steps of the previous section were repeated. As a nitrogen source, 0.08 g of ethylenediamine was added to the mixture, and then the pH was adjusted to 10 by adding sodium hydroxide (1 M). The other steps were repeated as in the previous section^26^.

### Preparation of gCN sheet

The bulk gCN powder was prepared via an modified heat-etching method and the melamine precursor in a muffle furnace according to the described method in the article^27^. In briefly; 5 g of melamine was heated at 520 °C for 2 h in static air with a heating rate of 10 °C min^−1^, and then the obtained yellow product was naturally cooled to room temperature.

### Preparation of ZnF nanoparticles (NPs)

ZnF NPs were synthesized according to the described method in the work Yao et al.^28^. In a beaker, 21 ml of double distilled water, 3 mmol Zn (NO_3_)_2_⋅6H_2_O and 6 mmol Fe(NO_3_)_3_⋅9H_2_O were added and the mixture was stirred. Then by adding sodium hydroxide solution (2 M) to the above solution, its pH was adjusted to about 12 and it was mixed for 25 min. The achieved solution was placed in a Teflon-lined stainless-steel reactor and maintained for 6 h at 100 °C. The resulting precipitates were separated by centrifuge, washed with deionized water (5 times), and dried at 70 °C for 24 h.

### Preparation of gCN-STO:N hybrid nanocomposites

The gCN-STO:N nanocomposites were synthesized typically through the following method: a certain amount of gCN nanosheets was added to the methanol solution and then dispersed by an ultrasonic device for 30 min. The as-prepared gCN suspension was added to synthesized STO:N NPs, and the obtained mixture was dispersed (30 min) and stirred (3 h) at room temperature. The resulting precipitates were filtered out, washed with ethanol and deionized water several times, and dried in a vacuum at 60 °C for 12 h. Finally, the gCN-STO:N nanocomposites were obtained^29^. The prepared samples contained different weight percentages of STO:N, and were named with the following codes: gCN-STO:N10%, gCN-STO:N20% and gCN-STO:N30%.

### Preparation of gCN-STO:N@ZnF nanocomposites

gCN-STO:N@ZnF nanocomposites with three different weight percentages of ZnF (10, 20, and 30 wt%) were synthesized by the following method: 1 g of gCN-STO:N powder was added to distilled water, and the reaction solution was placed in an ultrasonic bath for 30 min to obtain a homogeneously dispersed suspension. The prepared ZnF NPs (10, 20, and 30 wt%) were mixed with gCN-STO:N suspension for 30 min by sonication and then refluxed at 100 °C for 2 h. The suspension was cooled to room temperature. The resulting nanocomposite was filtered and dried in an oven at 80 °C. As a result, the prepared gCN-STO:N@ZnF nanocomposites were named gCN-STO:N@ZnF10%, gCN-STO:N@ZnF20%, and gCN-STO:N@ZnF30%, respectively.

### Ethical approval

1. This material is the authors' own original work, which has not been previously published elsewhere.
2. The paper is not currently being considered for publication elsewhere.
3. The paper reflects the authors' own research and analysis in a truthful and complete manner.

## Results and discussion

### Characterization

**Figure 1:**
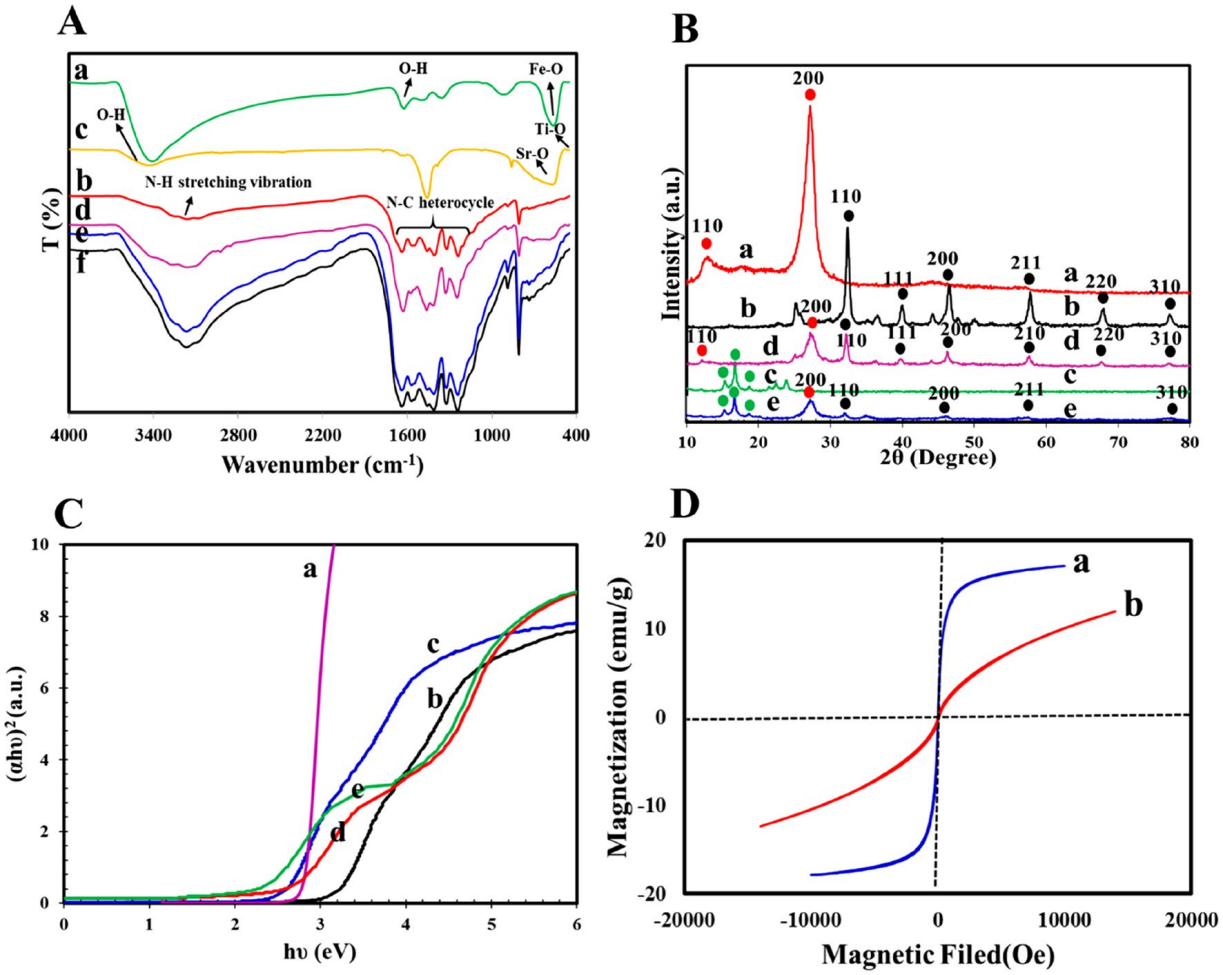
(**A**) FT-IR spectra of (a) ZnF NPs, (b) gCN, (c) STO:N, (d) gCN-STO:N, and (e) gCN-STO:N@ZnF, (f) gCN-STO:N@ZnF after degradation; (**B**) XRD spectra of (a) gCN, (b) STO:N, (c) ZnF NPs, (d) gCN-STO:N, and (e) gCN-STO:N@ZnF; (**C**) Tauc Plots for the (a) gCN, (b) STO, (c) STO:N, (d) gCN-STO:N, and (e) gCN-STO:N@ZnF; (**D**) VSM curves of (a) ZnF, and (b) gCN-STO:N@ZnF.

FT-IR analysis was used to determine the chemical bonds of the synthesized gCN, ZnF, STO:N, gCN-STO: N, and gCN-STO:N@ZnF samples, which the results are shown in Fig. 1A. According to the FT-IR spectra of the STO:N sample, the peak in the 450 cm^−1^ regions is related to the bonds of the stretching vibration of Ti–O, and the peak appearing in 600 cm^−1^ indicates the Sr–O bond of the STO:N. The peak in the 450 cm^−1^ regions is assigned to the N=O bond, which is associated with the impurity of N_2_. As shown in the figure, the spectrum of the 3400 cm^−1^ regions confirms the stretching vibration of OH^26^. In the FT-IR spectrum of the pure gCN sample, peaks in the region of 1247–1574 cm^−1^ correspond to the stretching vibration modes of the N–C heterocycle^30^. Also, the appearance of a broad peak at 3188 cm^−1^ is due to the N–H stretching vibration. In addition, the sharp peak in the 806 cm^-1^ regions represents the heptazine ring^31^. For ZnF nanoparticles, the stretching vibration modes of the Fe–O and O–H appeared in the range of 575 cm^−1^ and 1628 cm^−1^, respectively^32^. In addition, by loading STO:N and ZnF on gCN, all peaks belonging to STO:N and ZnF were observed in gCN-STO:N and gCN-STO:N@ZnF samples, which shows the photocatalysts are synthesized successfully.

The X-ray diffraction patterns of the as-prepared nanocomposites were investigated to identify the lattice planes, crystalline structure, phase purity, and crystallite size. Figure 1B exhibitions the XRD diffraction patterns of gCN, ZnF, gCN-STO:N, and gCN-STO:N@ZnF composites. The XRD spectra for the gCN sample showed two peaks at 2θ = 13.1° and 27.4° related to plates 110° and 200°, respectively^29^. On the other hand, the peaks related to STO:N in 2θ = 32.4°, 39.9°,46.6°, 57.8°, 67.8°, and 72.2°, which are related to pages 110, 111, 200, 211, 220, and 310, respectively. As can be seen from the Fig, the decrease in the intensity of STO:N perovskite peaks hybridized with GCN demonstrates a reduction in their crystallization in the samples of gCN-STO:N and gCN-STO:N@ZnF^29^. When ZnF was integrated over the gCN-STO:N, besides the characteristic peaks of gCN and STO:N, the other peaks at 2θ = 18.15°, 29.28°, 35.17°, 42.87°, 53.13°, 56.57°, and 62.89° can be attributed to the reflections of the (111(, (220(, (311(, (400(, (422(, (511) and (440) planes, respectively^33^.

The optical absorption properties of STO, STO:N, gCN, gCN-STO:N, and gCN-STO:N@ZnF samples were studied by UV–vis scattered reflectance spectroscopy. The bandgap values of the samples are determined by the Tauc method based on the relationship of tangent lines (ahυ)^2^ to energy (hυ)^12^. As evident in Fig. 1C, the estimated the bandgap values for the samples STO, STO:N, gCN, gCN-STO:N, and gCN-STO:N@ZnF are approximately 3.2, 2.58, 2.6, and 2.2 eV respectively, which correspond with the reported literature^26,34–39^.

VSM analysis was used to evaluate the magnetic properties of the ZNF NPs and gCN-STO:N@ZNF nanocomposites at room temperature, and the results are shown in Fig. 1D. Saturated magnetization for ZnF NPs and gCN-STO:N@ZNF nanocomposite, 15 and 5.15 emu/g were obtained, respectively. The comparison of these results confirms that the decoration of ZnF NPs with gCN and STO:N reduces the saturated magnetization of ZnF nanoparticles. But, the results indicate that the magnetic separability of the gCN-STO:N@ZNF nanocomposite can also be preserved and easily separated from the aqueous suspension using the external magnetic field.

**Figure 2:**
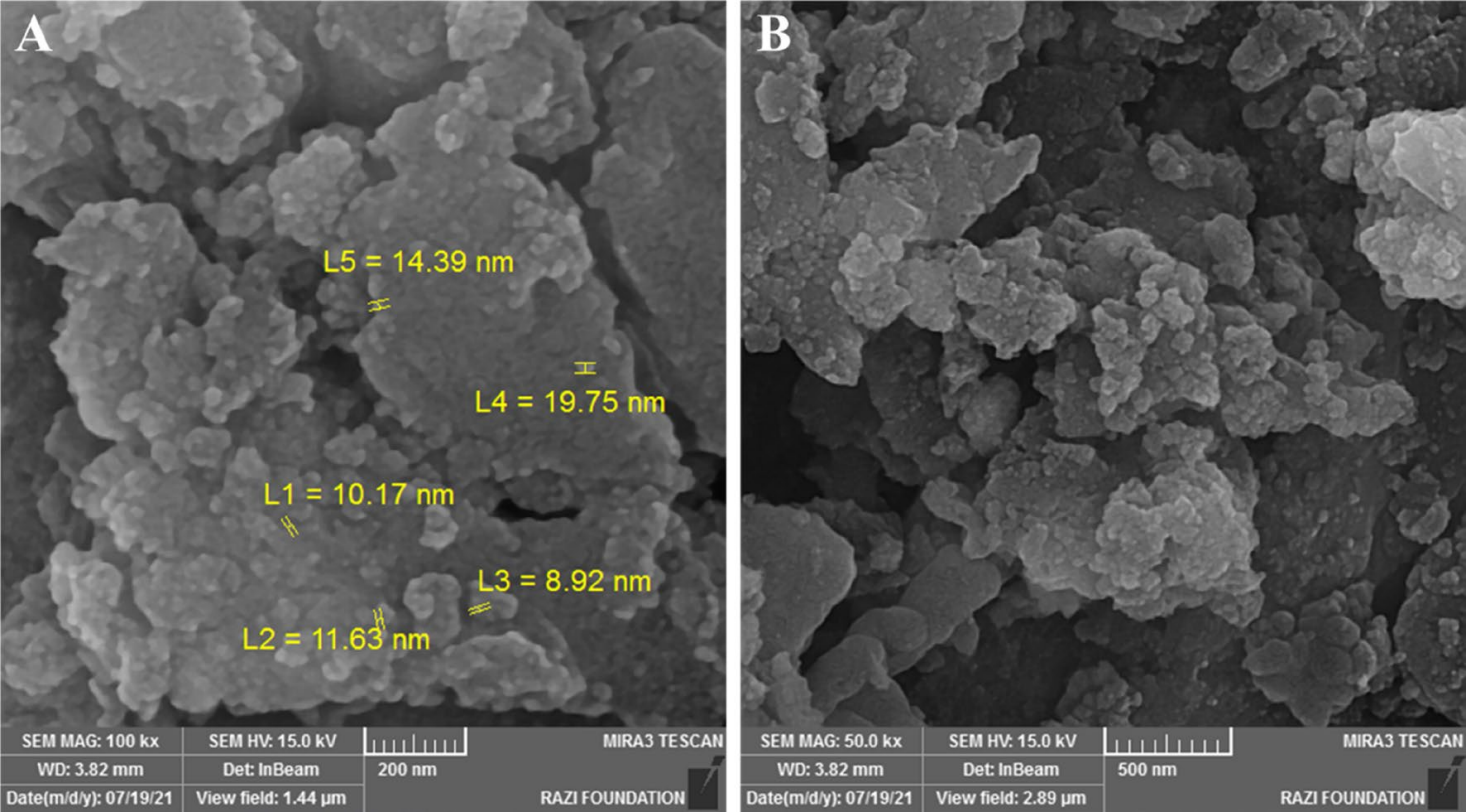
The SEM image of the gCN-STO:N@ZnF sample (**A**) before degradation, (**B**) after degradation.

**Figure 3:**
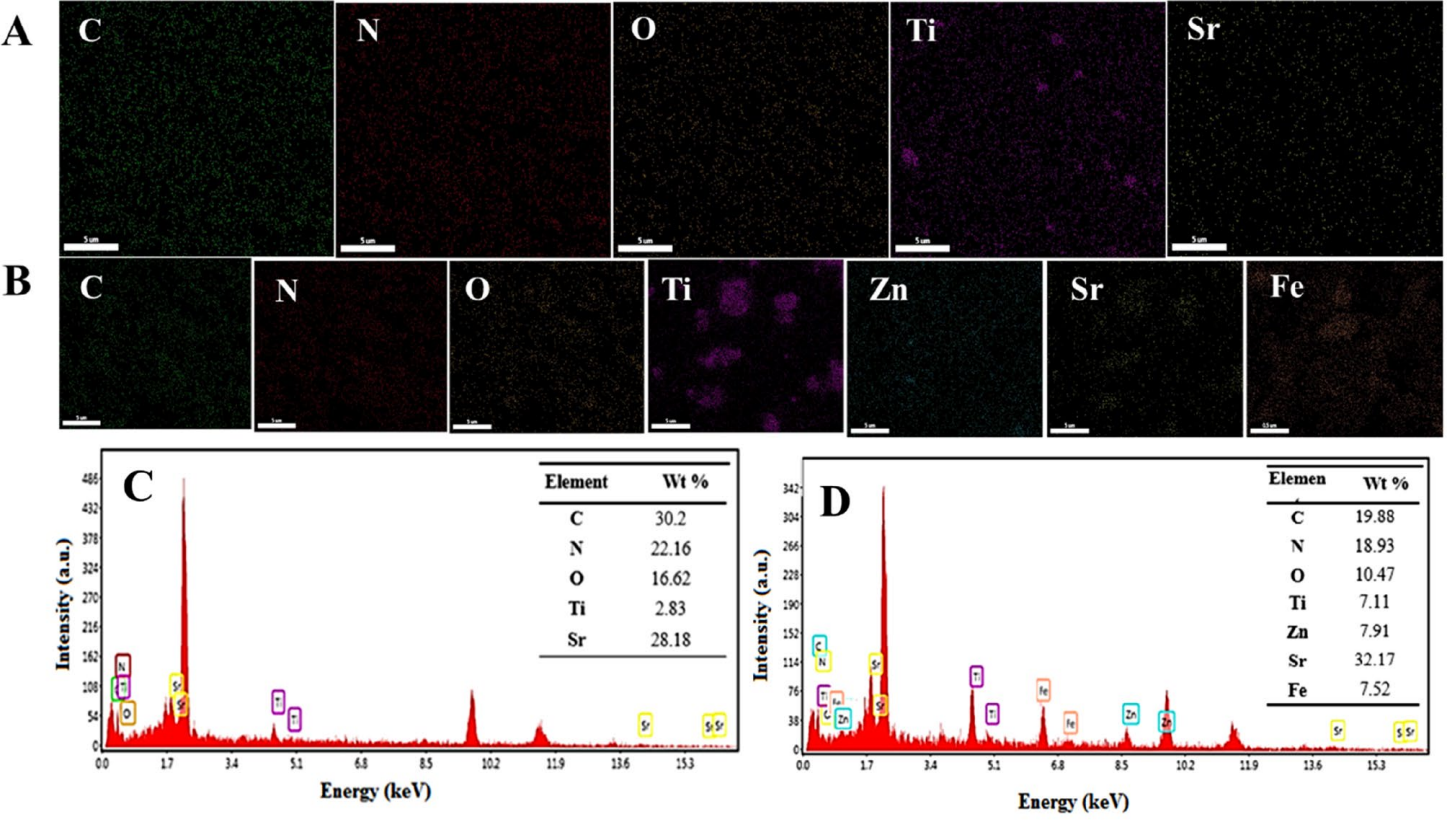
Element mapping of (**A**) gCN-STO:N sample, (**B**) gCN-STO:N@ZnF sample; EDX spectra of (**C**) gCN-STO:N sample, (**D**) gCN-STO:N@ZnF sample.

The morphology of the gCN-STO:N@ZnF sample before degradation and after degradation were exposed through SEM (Fig. 2) observations. After the degradation process, the structure of the photocatalysts has been preserved, which indicates the stability of these samples (Fig. 2B). Figure 3 depicted the EDX spectrum and elemental mapping of the gCN-STO:N and gCN-STO:N@ZnF. Based on the results N, C, Ti, Sr, Fe, and Zn elements were homogeneously distributed on the surface of the nanocomposite.

**Figure 4:**
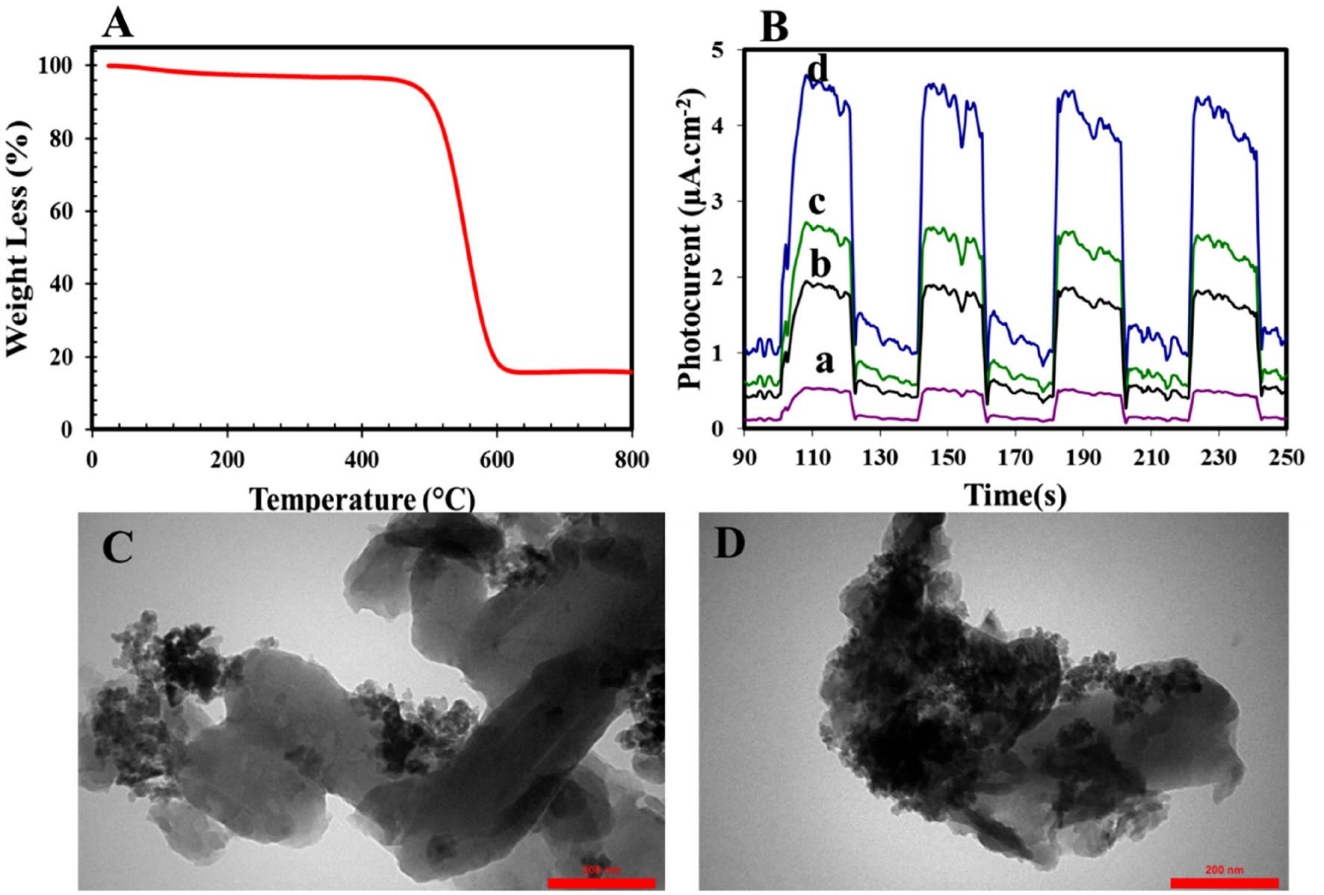
(**A**) TGA curve of gCN-STO:N@ZnF; (**B**) Photocurrent density of the (a) STO:N, (b) gCN, (c) gCN-STO:N and (d) gCN-STO:N@ZnF; TEM images of (**C**) gCN-STO:N and (**D**) gCN-STO:N@ZnF.

TGA analysis was used to study the thermostability of photocatalysts and evaluation the grafting percent of ZnF and STO:N over gCN, and the results are exhibited in Fig. 4A. According to the TGA curve, the thermal decomposition of gCN begins at 540 °C and is completed at 600 °C, which is attributed to gCN combustion because a large part of nanocomposites is gCN.

Optical and electronic properties can be mentioned among the factors affecting the performance of photocatalysts. In this work, to investigate the separation efficiency of photoinduced hole/electron pairs, photocurrent was measured and analyzed. As can be seen from Fig. 4B, the response of gCN-STO:N@ZnF20 was higher than gCN, STO:N, and ZnF NPs. It shows the increase in the high separation rate of electron–hole pairs with morphological control and the combination process.

The TEM results can be seen in Fig. 4C and D. According to Fig. 4C, the surfaces of gCN nanosheets are covered with STO:N NPs. Also, as seen in Fig. 4D, the presence of ZnF and STO:N NPs on the surface of gCN-STO:N@ZnF nanocomposite means the successful stabilization of these NPs on this nanocomposite.

**Figure 5:**
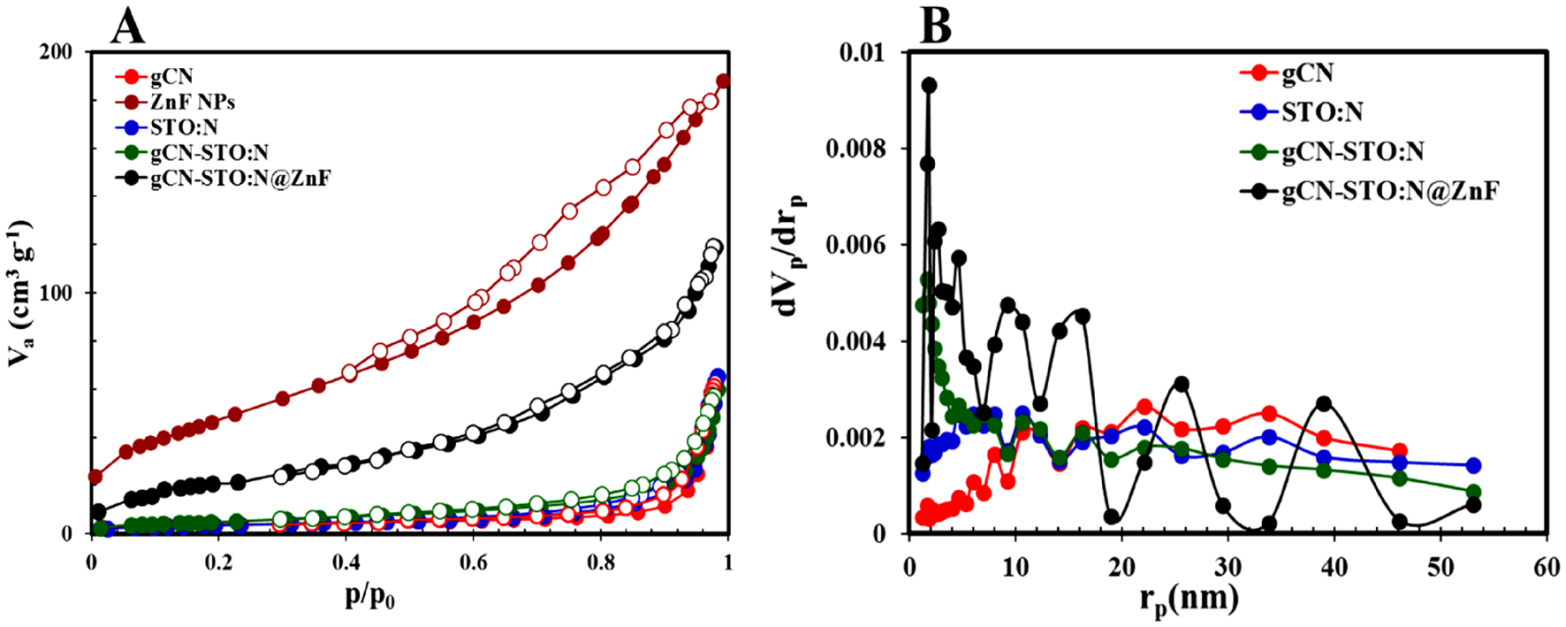
(**A**) Nitrogen adsorption–desorption isotherms, (**B**) BJH plots of the photocatalysts.

The textural properties, BET specific surface area and Barrett–Joyner–Halenda (BJH) of pure gCN, pure ZnF, pure STO:N, gCN-STO:N nanocomposite, and gCN-STO:N@ZnF nanocomposite were investigated using nitrogen adsorption analysis. Table 1 and Fig. 5 show the results of BET. As can be seen from the graphs, the samples show type III isotherms with H_3_ hysteresis loops that confirm the mesoporous nature of the synthesized photocatalysts. The ternary gCN-STO:N@ZnF nano-catalyst has the highest surface area. It is inferred that the remarkable photocatalytic efficiency of this nano-catalyst is attributed to the strong absorption of visible light. Furthermore, based on the BJH curve, the distribution of particles is in the range of 2 to 50 nm, so it can be an acceptable reason to confirm that the prepared photocatalysts are mesoporous.

**Table 1:** Textural properties of the photocatalysts.

Samples	Surface area (m^2^ g^−1^)	Mean pore diameter (nm)	Total pore volume (cm^3^ g^−1^)
gCN	13.672	24.592	0.09844
ZnF NPs	169.820	6.831	0.292
STO:N	29.051	24.592	0.101
gCN-STO:N	18.876	19.703	0.092
gCN-STO:N@ZnF	89.293	14.195	0.054

### Investigation of performance of photocatalysts under and kinetics of samples visible light

**Figure 6:**
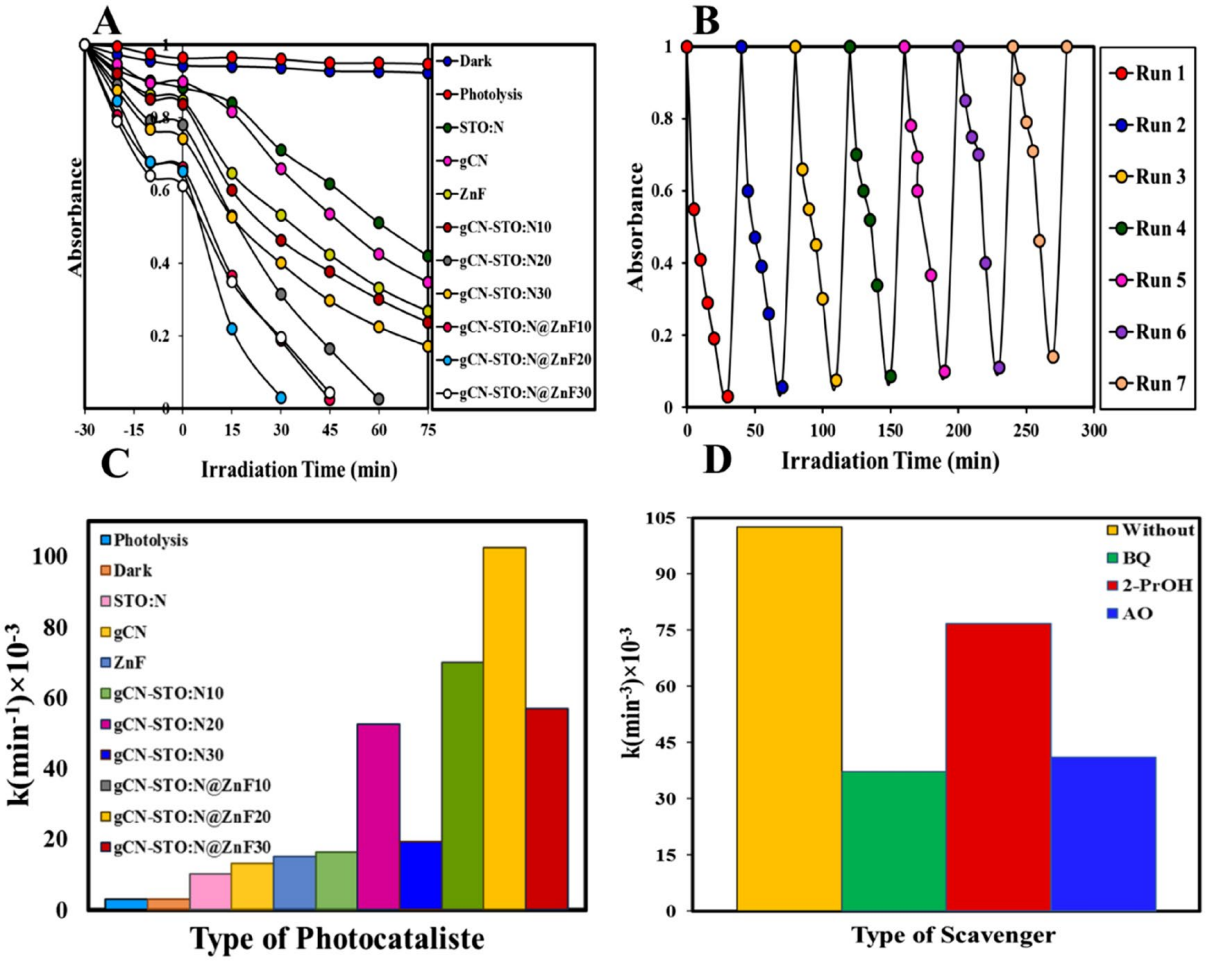
(**A**) Photodegradation of Rh-B by the synthesized samples; (**B**) Reusability of the gCN-STO:N@ZnF nanocomposite; (**C**) The degradation rate constant of Rh-B over the synthesized photocatalysts; (**D**) Constant rate of Rh-B degradation in the presence and absence of trappers.

Figure 6A shows the photocatalytic performance of samples synthesized for degradation of Rh-B (see raw date in Table S1). Degradation experimental were performed at pH = 7, initial concentration of 5 ppm, temperature of 25 °C and photocatalyst amount of 0.1 g. The increase in gCN NPs has improved the photocatalytic performance compared to STN. On the other hand, the integration of ZnF semiconductor over gCN-STO:N nanocomposite caused significantly improved photocatalytic performance in the degradation of Rh-B. It was observed that the gCN-STO: N photocatalyst shows the highest performance and destroys approximately 98% of Rh-B in 30 min. In addition, the Rh-B degradation kinetics curve was calculated for quasi-constant first-order kinetics, and the values of k are shown in Fig. 6C. The degradation rate constants on the gCN, ZnF, STO:N, gCN-STO:N20, and gCN-STO:N20@ZnF20 samples are 13.2 × 10^–3^, 15.2 × 10^–3^, 10.2 × 10^–3^, 52.6 × 10^–3^ min^−1^, and 102.6 × 10^–3^ min^−1^, respectively.

### The effect of scavengers

The role of active species by adding one mM of various scavengers, for example, 1,4-benzoquinone (BQ) as a quencher of radical (^⋅^O^−2^), 2-propanol (2-PrOH) as a quencher of hydroxyl radical (^•^OH), and ammonium oxalate as a quencher of holes (h^+^) were investigated^39–41^. The addition of 2-PrOH and BQ had a significant effect on the photocatalytic activity of the gCN-STO:N@ZnF nanocomposite. However, inhibitor AO had little effect on the photocatalytic activity of the gCN-STO:N20@ZnF nanocomposite. Therefore, the spaces of photocatalytic reactants during the Rh-B degradation process are as follows: ^⋅^O^2–^ > h^+^ > ^⋅^OH (Fig. 6D).

### Reusability of photocatalysts

The stability and regeneration of photocatalysts are significant parameters in their selection for use in the industry. Therefore, the reusability of the synthesized photocatalysts was studied and the results are reported in Fig. 6B (see raw date in Table S2). As the Fig. 6B shows, the gCN-STO:N@ZnF photocatalyst can be used for seven periods without losing its photocatalytic performance, so it can be said that it is a cost-effective photocatalyst.

### General mechanism of Rh-B degradation by gCN-STO: N@ZnF

**Figure 7:**
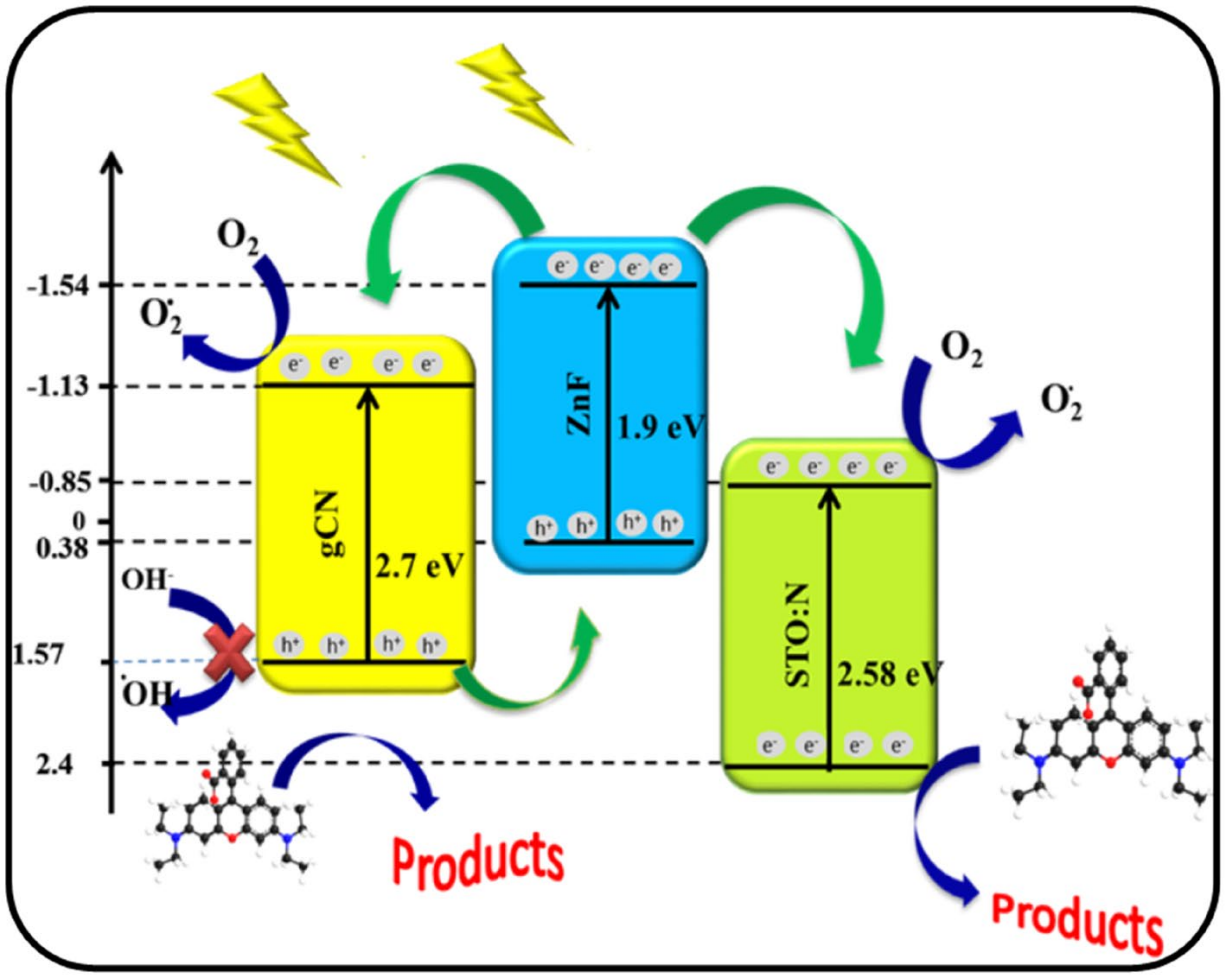
Probable mechanism of photocatalytic degradation of Rh-B by the gCN STO:N@ZnF photocatalyst under visible light illumination.

One of the most fundamental challenges of the photocatalytic process, which represents the production and enhancement of the e^-^/h^+^ pair separation, is the understanding of the possible mechanism. According to Fig. 7, STO:N has a lower conduction band edge and fermi level than gCN and ZnF. The amount of energy gap, conduction band potential, and valence band of photocatalysts are calculated according to empirical relations (1–3):

1 $${{\text{E}}}_{{\text{CB}}}=\upchi -{{\text{E}}}^{{\text{e}}}-{0.5{\text{E}}}_{{\text{g}}}$$

2 $${{\text{E}}}_{{\text{VB}}}={{\text{E}}}_{{\text{CB}}}+{{\text{E}}}_{{\text{g}}}$$

3 $$\upchi ={[\upchi {\left({\text{A}}\right)}^{{\text{a}}}\upchi {({\text{B}})}^{{\text{b}}}\upchi {({\text{C}})}^{{\text{c}}}]}^{1/({\text{a}}+{\text{b}}+{\text{c}})}$$

In these relationships, E_CB_, E_VB_, χ, E^e^ and E_g_ represent the conduction band potential, valence band potential, absolute electronegativity of the semiconductor, and the energy of free electrons in a vacuum (4.5 eV), and the energy gap, respectively^42^.

With the start of visible light irradiation, the prepared semiconductors are activated, and electrons are produced on the ZnF surface. These electrons are easily and quickly transferred from the conduction band of ZnF to the conduction band of STO:N and gCN, while the holes remain in the valence band. On the other hand, on the gCN surface, the holes are excited and transferred to the valence band of ZnF, thus leading to an increase in the e^−^/h^+^ separation. Subsequently, the electrons on the conduction band of STO:N and gCN are trapped by the adsorbed O_2_ and produce O_2_^⋅^ radicals. These radicals are highly reactive and can degrade organic dyes. Since the valence band potential of gCN is less positive relative to that of the potential, therefore, the produced ^•^OH radical species cannot be captured by using h^+^. Thus, the recombination of electrons and holes in the presence of STO:N is reduced and the photocatalytic performance is increased^43^. The generated O^2⋅−^ radicals and ^⋅^OH radicals attack the dye molecules adsorbed on the surface of the nanocomposite. These radicals oxidize the dye molecules, breaking them down into smaller, less harmful compounds. The proposed mechanism for this process is as follows:

$$ {\text{gCN-STO}}{:}{\text{N}}@{\text{ZnF}} + {\text{ h}}\upsilon \to {\text{gCN-STO}}{:}{\text{N}}@{\text{ZnF }}({\text{e}}^{ - }{_{{{\text{CB}}}}} + {\text{ h}}^{ + }{_{{{\text{VB}}}}} ) $$

$$ {\text{gCN-STO}}{:}{\text{N}}@{\text{ZnF }}({\text{e}}^{ - }{_{{{\text{CB}}}}} + {\text{ h}}^{ + }_{{{\text{VB}}}} )\left( {{\text{Transfer}}} \right) \to {\text{ZnF}}({\text{h}}^{ + }{_{{{\text{VB}}}}} ) \leftrightarrow {\text{gCN}}({\text{h}}^{ + }{_{{{\text{VB}}}}} ) $$

$$ {\text{STO}}{:}{\text{N}}({\text{e}}^{ - }{_{{{\text{CB}}}}} ) \, + {\text{ O}}_{{2}} \to \, {}^{ \cdot }{\text{O}}_{{2}}{^{ - }} $$

$$ {\text{STO}}{:}{\text{N}}({\text{e}}^{ - }_{{{\text{CB}}}} ) \, + {\text{ 2H}}^{ + } + {}^{ \cdot }{\text{O}}_{{2}}{^{ - }} \to {\text{H}}_{{2}} {\text{O}}_{{2}} + {\text{e}}^{ - } \to {}^{ \cdot }{\text{OH }} + {\text{ OH}}^{ - } $$

$$ {\text{ZnF}}({\text{h}}^{ + }{_{{{\text{VB}}}}} )  + {\text{ H}}_{{2}} {\text{O}}/{\text{ OH}}^{ - } \to {}^{ \cdot }{\text{OH}} $$

$$ {}^{ \cdot }{\text{OH }} + {\text{Rh-B}} \to {\text{ CO}}_{{2}} + {\text{H}}_{{2}} {\text{O }}\left( {\text{Degradation dye}} \right) $$

### Comparison of gCN STO:N@ZnF with photocatalysts studied in the other literature

The competitiveness of the currently developed catalyst has been examined in comparison to different catalysts mentioned in the literature for their Rh-B degradation efficiency, as illustrated in Table 2. Taking into account all the advantages and disadvantages of the reported photocatalyst systems, it is evident that the developed photocatalyst demonstrates in this research a high degradation efficiency compared to other systems.

**Table 2:** Comparative of photocatalysts for Rh-B degradation.

Photocatalyst	Reaction time (min)	Ref
gCN STO:N@ZnF	30	This study
10%MWCNT/TNT	60	^44^
V_2_O_5_/TiO_2_	70	^45^
g-C_3_N_4_/TiO_2_	78	^46^
α-MnO_2_/Pal	180	^47^
CoFe/SBA-15	95	^48^
NiO-NiFe_2_O_4_-rGO	100	^49^

## Conclusions

In this research, we successfully synthesized a novel ternary photocatalyst, highly efficient and magnetically reusable denoted as gCN-STO:N@ZnF, tailored for visible-light-driven applications. Comparative assessments of photocatalytic activity demonstrated that the gCN-STO:N@ZnF ternary nanocomposite outperforms the gCN-STO:N binary nanocomposite. Particularly, the gCN-STO:N@ZnF20 nanocomposite, as an advanced photocatalyst, exhibited superior efficiency in Rh-B degradation within just 30 min under visible light when compared to previously reported photocatalysts. The enhanced photocatalytic performance of our synthesized gCN-STO:N@ZnF nanocomposites under visible light irradiation can be attributed to their improved capacity for visible light absorption, effectively minimizing the recombination of electron–hole pairs and expanding the surface area. Furthermore, our study demonstrated that these photocatalysts can be reused for up to seven cycles without experiencing significant loss of degradation’s efficiency. In summary, our work has successfully introduced a novel ternary gCN-STO:N@ZnF photocatalyst that not only exhibits enhanced photocatalytic performance but also offers the added advantage of recyclability for the degradation of pollutants. Also, its ability to efficiently harness visible light and its magnetic properties make it a promising candidate for sustainable and effective photocatalytic applications. Overall, this study demonstrates the promising performance of the magnetic perovskite nanohybrid for the degradation of environmental pollutants, offering a potential solution for addressing environmental challenges.

### Supplementary Information

Supplementary Tables.

## Data Availability

All data generated or analysed during this study are included in this published article are available in the supplementary information file.
