# Supplementary material for: Magnetic perovskite nanohybrid based on g-C3N4 nanosheets for photodegradation of toxic environmental pollutants under short-time visible irradiation
Source: Sci Rep. 2023 Dec 3;13:21323. doi: 10.1038/s41598-023-48725-x (PMC10694144; doi:10.1038/s41598-023-48725-x)
Supplement: Supplementary file 1 — Supplementary Tables. [file 41598_2023_48725_MOESM1_ESM.docx]

**supplementary information**

**Magnetic Perovskite Nanohybrid Based on g-C_3_N_4_ Nanosheets for Photodegradation of Toxic Environmental Pollutants under Short-time Visible Irradiation**

Soheila Sharafinia^1^, Abdolhadi Farrokhnia*^1^, Ensieh Ghasemian Lemraski^2^, Alimorad Rashidi^3^

^1^Department of chemistry, Faculty of Science, Shahid Chamran University of Ahvaz, Ahvaz, Iran

^2^ Department of chemistry, Faculty of Science, Ilam University, Ilam, Iran

^3^ Nanotechnology Research Center, Research Institute of Petroleum Industry (RIPI), Tehran, Iran

*****Corresponding Author:

E-mail addresses**:** [farrokhnia@scu.ac.ir](mailto:farrokhnia@scu.ac.ir); [a**.**farrokhnia@gmail.com](mailto:a.farrokhnia@gmail.com)

**Table S1.** The data raw of photocatalytic performance of samples synthesized for degradation of Rh-B.

| **gCN-STO:N@ZnF30** | **gCN-STO:N@ZnF20** | **gCN-STO:N@ZnF10** | **gCN-STO:N30** | **gCN-STO:N20** | **gCN-STO:N10** | **STO:N** | **ZnF** | **gCN** | **Photolysis** | **Dark** |  |
| --- | --- | --- | --- | --- | --- | --- | --- | --- | --- | --- | --- |
| **Ads** | **Ads** | **Ads** | **Ads** | **Ads** | **Ads** | **Ads** | **Ads** | **Ads** | **Ads** | **Ads** | **Time (min)** |
| 1 | 1 | 1 | 1 | 1 | 1 | 1 | 1 | 1 | 1 | 1 | -30 |
| 0.789 | 0.845 | 0.805 | 0.874 | 0.89 | 0.92 | 0.931 | 0.917 | 0.947 | 0.994 | 0.973 | -20 |
| 0.639 | 0.678 | 0.676 | 0.768 | 0.791 | 0.85 | 0.9 | 0.862 | 0.895 | 0.974 | 0.955 | -10 |
| 0.6121 | 0.652 | 0.662 | 0.742 | 0.779 | 0.837 | 0.881 | 0.847 | 0.898 | 0.963 | 0.942 | 0 |
| 0.348 | 0.22 | 0.365 | 0.527 | 0.53 | 0.6 | 0.84 | 0.646 | 0.815 | 0.965 | 0.94 | 15 |
| 0.195 | 0.03 | 0.188 | 0.4 | 0.315 | 0.463 | 0.71 | 0.531 | 0.659 | 0.96 | 0.936 | 30 |
| 0.043 |  | 0.025 | 0.297 | 0.164 | 0.376 | 0.618 | 0.423 | 0.535 | 0.95 | 0.927 | 45 |
| 1 |  |  | 0.225 | 0.027 | 0.3 | 0.51 | 0.332 | 0.424 | 0.95 | 0.926 | 60 |
|  |  |  | 0.172 |  | 0.237 | 0.419 | 0.268 | 0.347 | 0.947 | 0.922 | 75 |
|  |  |  |  |  | 0.188 | 1 | 0.22 | 0.28 | 0.937 | 0.915 | 90 |
|  |  |  |  |  |  | 0.931 | 0.244 | 0.277 |  |  | 120 |

**Table S2.** The data raw of reusability of the gCN-STO:N@ZnF nanocomposite for degradation of Rh-B.

| **Run 7** | **Time (min)** | **Run 6** | **Time (min)** | **Run 5** | **Time (min)** | **Run 4** | **Time (min)** | **Run 3** | **Time (min)** | **Run 2** | **Time (min)** | **Run 1** | **Time (min)** |
| --- | --- | --- | --- | --- | --- | --- | --- | --- | --- | --- | --- | --- | --- |
| 240 | 1 | 200 | 1 | 160 | 1 | 120 | 1 | 80 | 1 | 40 | 1 | 0 | 1 |
| 245 | 0.91 | 205 | 0.85 | 165 | 0.78 | 125 | 0.7 | 85 | 0.66 | 45 | 0.6 | 5 | 0.55 |
| 250 | 0.79 | 210 | 0.75 | 170 | 0.694 | 130 | 0.6 | 90 | 0.55 | 50 | 0.47 | 10 | 0.41 |
| 255 | 0.71 | 215 | 0.7 | 170 | 0.6 | 135 | 0.52 | 95 | 0.45 | 55 | 0.39 | 15 | 0.29 |
| 260 | 0.462 | 220 | 0.4 | 180 | 0.366 | 140 | 0.338 | 100 | 0.3 | 60 | 0.26 | 20 | 0.19 |
| 270 | 0.14 | 230 | 0.11 | 190 | 0.1 | 150 | 0.087 | 110 | 0.075 | 70 | 0.056 | 30 | 0.031 |
| 280 | 1 | 240 | 1 | 200 | 1 | 160 | 1 | 120 | 1 | 80 | 1 | 40 | 1 |
